# A Versatile Orthotopic Nude Mouse Model for Study of Esophageal Squamous Cell Carcinoma

**DOI:** 10.1155/2015/910715

**Published:** 2015-03-05

**Authors:** Joseph Chok Yan Ip, Josephine Mun Yee Ko, Valen Zhuoyou Yu, Kwok Wah Chan, Alfred K. Lam, Simon Law, Daniel King Hung Tong, Maria Li Lung

**Affiliations:** ^1^Department of Clinical Oncology, University of Hong Kong, Hong Kong; ^2^Department of Pathology, University of Hong Kong, Hong Kong; ^3^Cancer Molecular Pathology, Griffith Medical School and Menzies Health Institute Queensland, Griffith University, Gold Coast, QLD 4222, Australia; ^4^Department of Surgery, University of Hong Kong, Hong Kong; ^5^Center for Cancer Research, University of Hong Kong, Hong Kong

## Abstract

Increasing evidence indicates tumor-stromal interactions play a crucial role in cancer. An *in vivo* esophageal squamous cell carcinoma (ESCC) orthotopic animal model was developed with bioluminescence imaging established with a real-time monitoring platform for functional and signaling investigation of tumor-stromal interactions. The model was produced by injection of luciferase-labelled ESCC cells into the intraesophageal wall of nude mice. Histological examination indicates this orthotopic model is highly reproducible with 100% tumorigenesis among the four ESCC cell lines tested. This new model recapitulates many clinical and pathological properties of human ESCC, including esophageal luminal stricture by squamous cell carcinoma with nodular tumor growth, adventitia invasion, lymphovascular invasion, and perineural infiltration. It was tested using an AKT shRNA knockdown of ESCC cell lines and the *in vivo* tumor suppressive effects of AKT knockdown were observed. In conclusion, this ESCC orthotopic mouse model allows investigation of gene functions of cancer cells in a more natural tumor microenvironment and has advantages over previous established models. It provides a versatile platform with potential application for metastasis and therapeutic regimen testing.

## 1. Introduction

Esophageal cancer (EC) is a devastating cancer with a five-year survival ranging from 15% to 25% [1, 2]. It is ranked sixth in cancer mortality and eighth for cancer incidence worldwide [2–4]. Esophageal cancers are classified into two main histological subtypes, namely, esophageal squamous cell carcinoma (ESCC) and esophageal adenocarcinoma (EAC). ESCC comprises over 90% of esophageal cancers worldwide [5–8], but EAC is rapidly becoming the predominant histological type of EC in Australia, UK, US, and western European countries [9, 10]. The cancer shows a wide geographical variation with the highest prevalence region, termed the “Asian esophageal cancer belt,” including Turkey, northeastern Iran, southern and eastern Africa, and certain regions of northern and central China such as Henan and Shanxi [2, 11, 12]. The majority of the patients are diagnosed at advanced metastatic stages with poor clinical outcomes [1, 2, 4]. Biomarkers for prevention, early disease detection, prognostication of poor disease outcome, and guided therapeutic treatment options are necessary to improve survival outcomes.

Cancer development is a complex multistep process [13–15]. Accumulation of genetic alterations leads to deregulation of the normal intracellular signaling network and interactions with the extracellular matrix environment, which are important factors associated with cancer development [13–16]. The tumor microenvironment and its interactions with the tumor play a crucial role in tumor growth dynamics. The rationale to establish an orthotopic ESCC model is to recapitulate more closely the microenvironment of the tumor in its organ of origin. Establishment of orthotopic models for cancers in different organs has been the preferred choice for cancer studies due to the unique tumor microenvironments provided at different organ sites. Orthotopic animal models provide the best fidelity for recapitulation of the tumor microenvironment, which are invaluable for cancer and drug development studies [16–23].

In ESCC, a limited number of orthotopic models have been established, but all have some shortcomings. The currently available models are not well suited for functional and signaling studies of tumor-stromal interactions and metastasis in ESCC. Three previously established EC orthotopic models involved (1) surgically binding small pieces of subcutaneous tumors to a mechanically damaged esophagus, (2) inoculating cancer cells into the esophagus with matrigel without any visual aids to determine actual sites inoculated, and (3) inoculating the tumor cells into the esophageal wall through a hole in the stomach near the gastroesophageal junction [24–26]. We have an interest in the study of the functional properties and signaling pathways of tumor suppressor genes and other candidate genes involved in ESCC cancer development. These tumor-suppressive and antimetastatic functions are heavily influenced by tumor-stromal interactions in ESCC. Thus, we developed an ESCC orthotopic model with pathological features highly mimicking human ESCC tumors and allowing such questions to be addressed.

ESCC is reported to occur mainly in the lower two-thirds of the esophagus with between 58.3% and 66% occurring at the middle esophagus and approximately 26% to 38.9% located in the lower third of the esophagus [27, 28]. Hence, we developed an ESCC orthotopic model using luciferase-labeled cell lines targeting near the middle esophagus close to the diaphragm and away from the esophageal-stomach junction of the esophagus of the nude mouse. This model allows reproducible tumor formation and real-time imaging of the tumor progression. We verified the practicality of this system by studying the changes in a molecular pathway utilizing an AKT (protein kinase B) shRNA knockdown approach in ESCC cell lines to knockdown AKT, which is frequently deregulated in cancers, to confirm its functionality in this* in vivo* animal model system.

## 2. Materials and Methods

### 2.1. ESCC Cell Lines

Four luciferase-labelled ESCC cell lines, 81-T [29], KYSE30 [30], KYSE150 [30, 31], and SLMT-1 [32], were used for* in vitro* and* in vivo* studies. The cell lines were authenticated by the AmpF*ℓ*STR Identifier PCR Amplification kit (Life Technologies, Carlsbad, CA, USA). The 81-T, KYSE30, KYSE150, and SLMT-1 cells were labelled with luciferase [33] and were cultured as previously described [34].

### 2.2. Orthotopic Model

BALB/cAnN-nu mice at four to five weeks of age were obtained from the Laboratory Animal Unit at the University of Hong Kong. The orthotopic inoculation protocol was approved by the Committee on the Use of Live Animals in Teaching and Research. Animals were housed in the Laboratory Animal Unit facility accredited by the Association of Assessment and Accreditation of Laboratory Animal Care International. The environment was kept between 16°C and 26°C with relative humidity between 30 and 70% under a regular 12-hour light, 12-hour dark cycle. Before surgery, each mouse was anesthetized by intraperitoneal injection of ketamine/xylazine (100 mg/kg and 10 mg/kg). Mice were placed ventrally on the heating pad with the small animal surgery system (Braintree Scientific, Braintree, MA, USA). Disinfection with iodophors followed by 70% ethanol was applied on the intended incision area below the left side of the rib cage and diaphragm near the stomach prior to making the incision. A skin incision no larger than one cm was made, followed by an aligned peritoneal dissection. Retractors were used to keep the incisions open for better visualization. Connective tissues between the small left liver lobe and the esophagus were carefully separated. The stomach was gently pulled out caudally to create adequate tension to straighten the esophagus for injection of cells. ESCC cells in 10 *μ*L plain medium were inoculated with a 30 G 1 cc insulin syringe into the muscularis externa under a dissecting microscope, SMZ445 (Nikon, Tokyo, Japan). Cell suspensions were loaded to the syringe one by one for each mouse after the cell suspension was mixed well and measured with a Pipetman to ensure the consistency of the cell concentration and volume delivered. Upon observing edema forming at the inoculation site, a cotton swab was gently pressed on the inoculation site while removing the needle. This prevents any leakage of cells to the peritoneal cavity and is a preventive measure against peritoneal carcinomatosis. Vicryl absorbable sutures were used to close the peritoneum wound. Outer skin incision was closed with the reflex wound clips. Animals were warmed by a heat lamp or heating pad until fully regaining motility. Animal welfare was monitored as assessed by body weight, hydration status, activity status, appetite, and behavior.

### 2.3. Live Imaging

Live imaging was done weekly by using the Xenogen* in vivo* imaging system, IVIS-100 (Perkin Elmer, MA, USA) to monitor the orthotopic tumor growth kinetics of the luciferase-labelled ESCC cell lines injected into the mice and to observe for metastasis. The 3D live images were captured by using the Xenogen IVIS Spectrum. Luciferin substrate (Perkin Elmer) at 150 mg/kg was injected into the animals prior to bioluminescence imaging. Animals were euthanized at the end of the study at weeks 3 to 5 to excise the orthotopic tumors. The tumors were dissected and fixed in formalin and embedded in paraffin. Sectioned tissues were stained with hematoxylin and eosin for histological examination by pathologists (Kwok Wah Chan and Alfred K. Lam).

### 2.4. Subcutaneous Nude Mouse Tumorigenicity Assay

For the subcutaneous injection, 1 × 10^6^ ESCC tumor cells resuspended in 100 *μ*L were inoculated into the flanks of the animals, as previously described using the BALB/cAnN-nu mice [34].

### 2.5. AKT Knockdown Constructs

The AKT knockdown oligonucleotides were cloned into a pLKO.1 cloning vector (AddGene number 10878). The AKT knockdown oligonucleotide sequences were obtained from The RNAi Consortium/Public TRC portal (http://www.broadinstitute.org/rnai/public/), targeting sequence 984 (construct ID: TRCN0000288787) and sequence 1793 (TRCN0000199454) on AKT. A scrambled sequence (AddGene number 1864) was used as a control. Results from the two knockdown oligos were pooled and analyzed against that of controls.

### 2.6. *In Vitro* Real-Time Invasion Assay

The real-time cell invasion assay was performed by using the ACEA Xcelligence RTCA system with CIM plate as previously described [35, 36].

### 2.7. Western Blot Analysis

Western blot analysis was performed to verify efficient knockdown of AKT expression with AKT (Cat number 9272, Cell Signaling, Beverly, MA, USA) and p84 (Cat number GTX70220, Genetex, Irvine, CA, USA) was used as a loading control. Cell lysate collection and protein purification were performed as previously described [35].

### 2.8. Quantitative-PCR (Q-PCR)

Q-PCR was performed with SYBR-green PCR core reagent kits (Applied Biosystems, CA, USA) in a Step-One Plus machine (Applied Biosystems, CA, USA) and the primer sequences of IL8 and VEGFA were as previously described [37].

### 2.9. Statistical Analysis

Numerical data were analyzed by the *t*-test, whereas the categorical data were studied by either Fisher's exact test or Chi-square test.

## 3. Results

### 3.1. Establishment of ESCC Orthotopic Tumors Monitored by Live Animal Imaging

**Figure 1:**
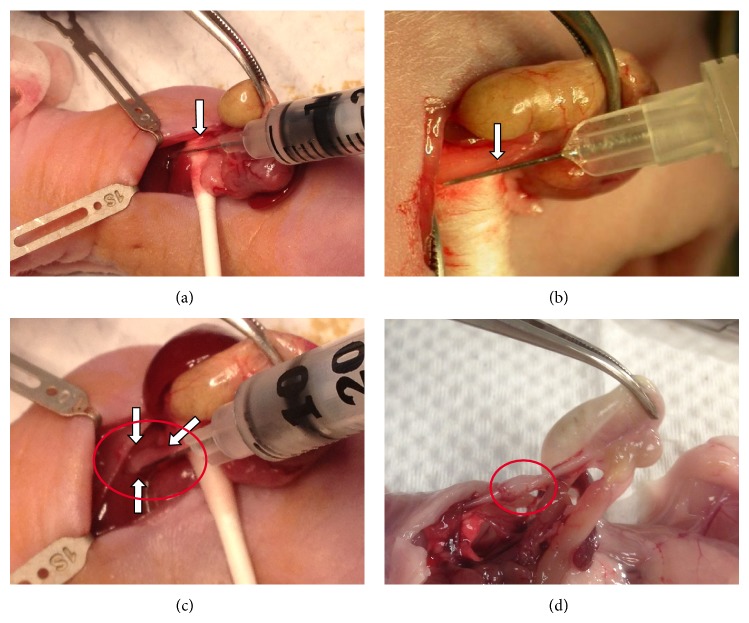
Surgical inoculation. (a) The needle insertion point near the middle esophagus below the diaphragm away from the esophagogastric junction is indicated by the arrow. (b) A thin layer of tissues covering the needle can be observed on the left side indicated by the arrow after inserting the needle into the esophageal wall. (c) The edema in the esophagus forming after successful inoculation of cancer cells is marked by circle and arrows. (d) A stretched esophagus with a solid orthotopic tumor is observed just below the diaphragm.

An ESCC orthotopic tumor model recapitulating multiple histopathological characteristics of human ESCC tumors was established by inoculating ESCC cancer cells into the muscularis externa of the esophageal wall near the middle esophagus below the diaphragm away from the esophagogastric junction (Figure 1(a)). Use of a dissecting microscope ensures that the needle was inserted underneath the thin membrane of the esophageal wall (Figure 1(b)). When the cells are inoculated into the muscularis externa, edema can be observed as an indication of successful inoculation (Figure 1(c)). If the cells were injected into the lumen, edema would be absent. A representative orthotopic solid tumor developing after inoculation with ESCC cells was observed near the middle esophagus below the diaphragm at the end-point of the study (Figure 1(d)).

**Figure 2:**
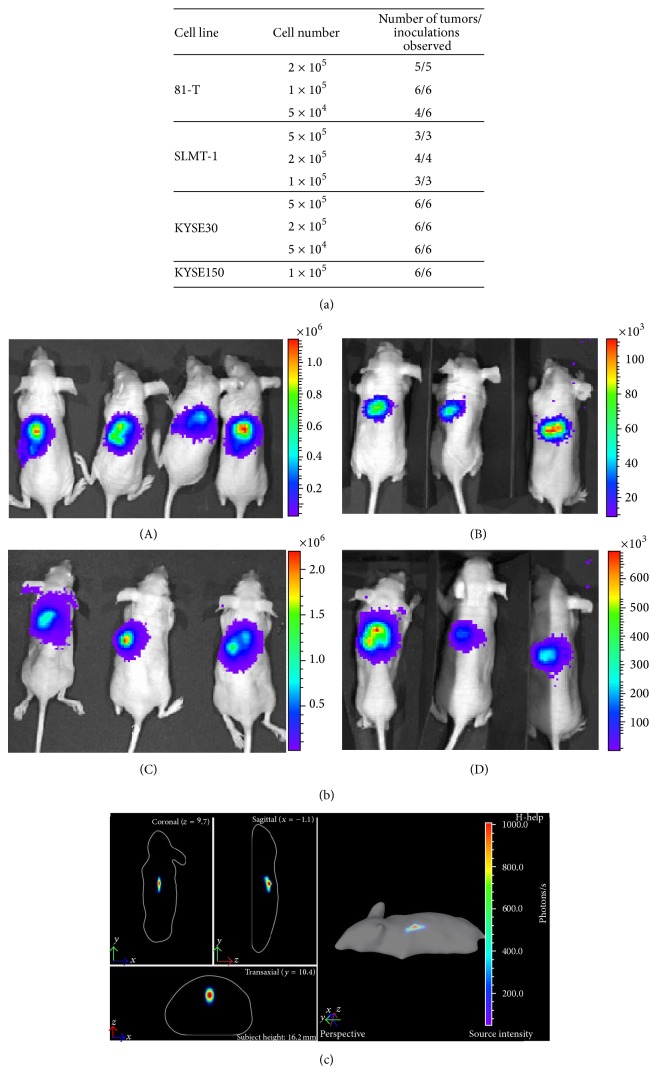
Orthotopic ESCC cell line model. (a) Different cell numbers were evaluated for orthotopic tumor formation for four ESCC cell lines. (b) Xenogen images of 10^5^ cells for four ESCC cell lines expressing luciferase illustrate the growth of the tumor with (A) KYSE150 luc, (B) KYSE30 luc, (C) 81-T luc, and (D) SLMT-1 luc. (c) The bioluminescence live animal images displaying coronal, sagittal, and transaxial cross-section views of the animal and providing tumor depth and location information of a KYSE150 orthotopic tumor in 3D taken by the Xenogen IVIS Spectrum.

Cell numbers ranging between 5 × 10^4^ and 5 × 10^5^ for 81-T, SLMT-1, KYSE30, and KYSE150 ESCC cell lines were tested in this orthotopic model (Figure 2(a)). The cell number range utilized was determined based on previous subcutaneous tumorigenicity assay results and adjusted after the pilot studies. Most tested cell lines produced 100% orthotopic tumors using the various cell numbers tested. The optimum cell concentrations for producing orthotopic tumors with consistent tumor sizes and survival times for mice, as well as being 100% tumorigenic for the tested ESCC cell lines, KYSE150 luc and SLMT-1 luc, are 1 × 10^5^ cells and for KYSE30 luc and 81-T luc are 2 × 10^5^ cells, each in a volume of 10 *μ*L. However, a trend of earlier death after injection of higher number of cells was observed (data not shown). When inoculated with higher cell numbers, a small number of the animals died within two weeks, while the majority needed to be euthanized by three weeks due to animal welfare issues. When inoculated with lower numbers of cells, the survival time was generally prolonged by one to two weeks depending on the cell line. Representative live animal images showed the high intensity of luciferin signals for each cell line in two dimensions (Figure 2(b)). 3D images can be obtained by using the Xenogen Spectrum, which produces more detailed images to visualize the depth and shape of the tumor (Figure 2(c)).

### 3.2. Characterization of* In Vivo* Orthotopic Tumors

**Figure 3:**
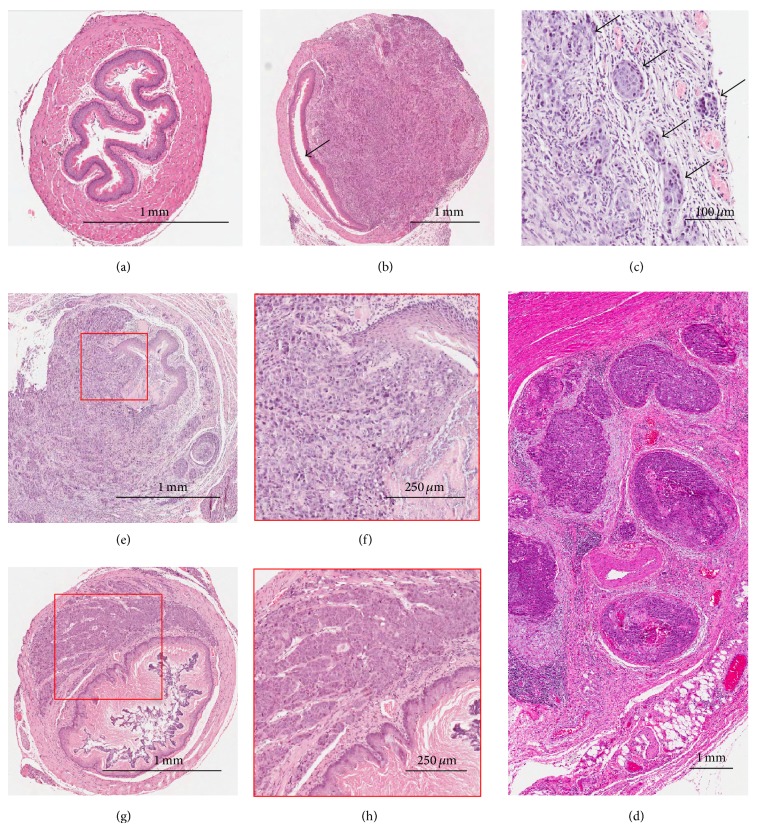
Hematoxylin- and eosin-stained (H&E) tissue images. (a) Cross section image of a naïve mouse esophagus. (b) Image of a mouse esophagus inoculated with KYSE150 showing esophageal luminal stricture due to tumor growth indicated by arrow. (c) H&E image of a KYSE150 orthotopic tumor containing multiple tumor nodules (indicated by arrows) with lymphovascular invasion. (d) Image of multiple tumor nodules in human ESCC, similar to what is observed in the mouse orthotopic model shown in (c). (e) Image of poorly differentiated KYSE150 orthotopic tumor indicates invasion from mucosa to adventitia and with lymphovascular permeation in the mouse esophagus at week 4. (f) A magnified view of the tumor invasion edge as indicated in the red-boxed area in (e) showing ulceration in lumen resulting from tumor invasion. (g) A cross-section H&E image of a mouse esophagus inoculated with SLMT-1. Orthotopic tumor is of low histological grade. (h) A magnified view of red-boxed area reveals large tumor nest near the lumen.

In order to gain insights for the extracellular matrix interaction between ESCC tumor cells and the stromal environment in the esophagus, the paraffin-embedded orthotopic ESCC tumors were carefully examined microscopically and histologically by pathologists (Kwok Wah Chan and Alfred K. Lam) after sectioning and staining with hematoxylin and eosin (H&E). Representative H&E images from histopathological examinations of the normal esophagus and orthotopic tumors are shown in Figure 3. Cross sections of the normal mouse esophagus (Figure 3(a)) and the esophagus after inoculation with KYSE150 showing luminal stricture due to the tumor growth (Figure 3(b)) are shown. Lymphovascular permeation is detected. The lesions in the animals (Figure 3(c)) were similar to those observed in human clinical samples (Figure 3(d)) and appeared as nodular growths. The results indicate the tumor cells were consistently inoculated in the muscularis externa of the mouse esophagus, which is where tumor cells are usually observed in the clinical ESCC samples. The orthotopic tumors derived from the KYSE150 cell line, a poorly differentiated ESCC cell line, show invasion from mucosa to adventitia and ulceration in the lumen of the mouse esophagus (Figures 3(e) and 3(f)). Other common characteristics in ESCC [38], including squamous differentiation and focal keratinization are also observed in orthotopic tumors derived from KYSE150. Representative H&E staining of the low histological grade orthotopic tumors derived from another ESCC cell line, SLMT-1, is shown in Figure 3(g). A magnified view of a large tumor nest near the lumen of the red-boxed area (Figure 3(h)) shows a less invasive tumor edge with the nearby tissue, compared to that derived from KYSE150 (Figure 3(f)).

### 3.3. Use of the ESCC Orthotopic Model for ESCC Functional and Signaling Studies Using a shRNA AKT Knockdown Approach

**Figure 4:**
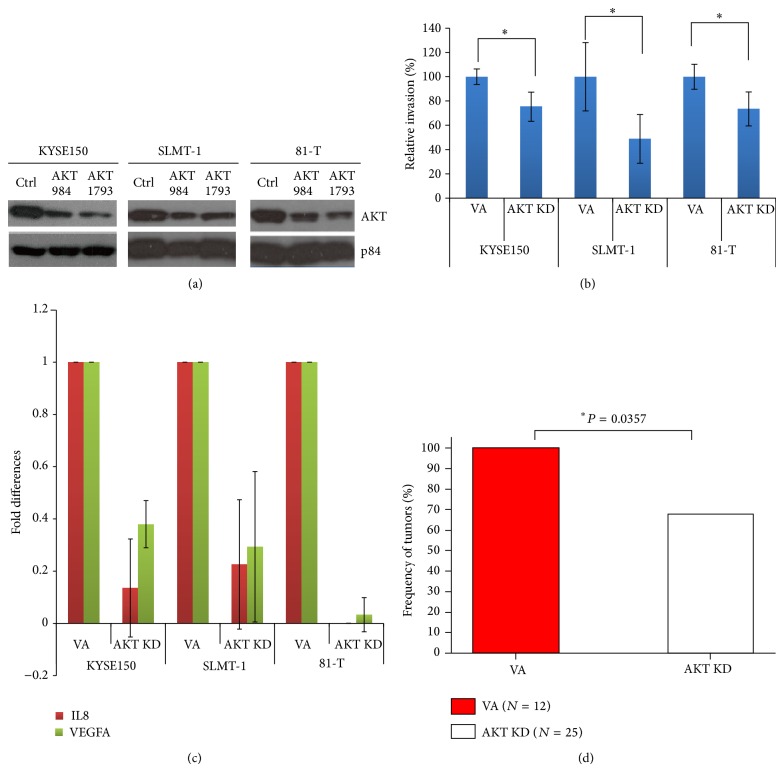
AKT knockdown. (a) AKT knockdown (KD) Western blots of three ESCC cell lines indicates successful AKT knockdown. (b)* In vitro* real-time invasion assays show significant inhibition in all three ESCC cell lines between vector alone (VA) and AKT KD; ∗ indicates *P* < 0.05. (c) Quantitative PCR data shows inhibition in AKT downstream angiogenesis markers, IL8 and VEGFA, in all three ESCC cell lines after AKT KD. (d) Histogram of incidence take rate, which is in terms of detectable luciferin signals after defined exposure taken at medium field in one minute, of the orthotopic assay demonstrates significant tumor growth inhibition comparing the combined AKT KD groups (17/25, 68%) versus the control (12/12, 100%) (*P* = 0.0357). Control (ctr) = scrambled oligonucleotide control.

Demonstration of the usefulness of the current established ESCC orthotopic model for ESCC studies was shown by shRNA AKT knockdown of this well-known oncogenic signaling pathway for driving cancer. Both AKT knockdown (KD) oligonucleotides at positions 984 and 1793 showed high efficiency reduction of AKT expression in the tested ESCC cell lines, KYSE150, SLMT-1, and 81-T (Figure 4(a)). The* in vitro* real-time invasion assay provided further functional evidence for the successful silencing effect of the AKT KD cell lines, which showed a significantly lower invasiveness, when compared with the scrambled control (Figure 4(b)). QPCR data provided additional molecular evidence for the effectiveness of the signaling cascade of AKT silencing. Downregulation of well-studied AKT downstream invasion-associated markers including IL8 and VEGFA [39–41] in the AKT knockdown cell lines was detected, as compared to the control (Figure 4(c)). Animals were inoculated with orthotopic tumors of control and AKT KD cells. The* in vivo* orthotopic tumor growth was monitored by bioluminescence. The AKT KD groups (17/25, 68%) versus the control (12/12, 100%) showed significant tumor growth inhibition (*P* = 0.0357) (Figure 4(d)).

## 4. Discussion

We have established a highly reproducible orthotopic animal model for surgical inoculation of ESCC cells into nude mice. In contrast to the esophageal adenocarcinomas, which are reported to arise from gastric cardia near the squamocolumnar junction and esophagogastric junction in the lower third of the esophagus [42, 43], ESCC is most commonly observed in the lower two-thirds of the esophagus [27]. In Hong Kong approximately 66% of the squamous cell carcinomas arise in the middle region of the esophagus [44]. In Asian populations, the majority of the esophageal cancers are squamous cell carcinomas. Thus, this current animal model focused on inoculating the ESCC cells directly near the middle portion of the esophagus close to the diaphragm, away from the squamocolumnar junction and esophageal-stomach junction, since the esophageal-stomach junction is where the majority of the esophageal adenocarcinomas arise [42, 43]. In this study, inoculation of cancer cells was done surgically into the esophagus directly with the aid of a dissecting microscope to ensure precise delivery of the cells into the esophageal wall. Utilizing an injection volume of only 10 *μ*L cells minimizes backpressure and leakage. The ESCC cell lines utilized in this study were engineered and labeled with luciferase to allow use of a more sensitive* in vivo* imaging system for detection of the tumor growth and metastasis.

The current established model now provides a reliable and versatile ESCC* in vivo* model platform with advantages over the previously established ESCC orthotopic models using implantation of subcutaneous ESCC tumors onto a mechanically damaged esophagus [25], inoculation via the oral route [26], or injection of ESCC cells into the submucosal layer through a hole in the stomach [24]. These earlier orthotopic models each have limitations. Surgically binding pieces of subcutaneous tumor to the esophagus can lead to the outgrowth of the implanted tumor cells outside the esophagus, invasion of surrounding tissues, and possible obstruction of the trachea and other structures in the animals. Our experience following the described nonsurgical method of inoculation of a mixture of matrigel and cells into the esophagus of the animal through the oral cavity resulted in tumor growth occurring outside the esophagus and finally developing esophageal luminal restricture from the external tumor due to the uncertainty of the insertion site [26]. For the surgical method, the inoculum volume of cancer cells was critical for successful formation of orthotopic tumors. The inner (luminal) surface of the mouse esophagus consists of keratinized stratified squamous epithelial cells, where the thickness is estimated to be approximately 3–5 layers of cells [45]. We found that leakage of tumor cells into the lumen with different inoculum volumes ranging from 20 *μ*L to 200 *μ*L and without the aid of a dissecting microscope for guiding inoculation in such constricted space was inevitable. The insertion site was also a critical factor affecting the eventual tumor formed. Tumors occasionally extended from the lower esophagus to the stomach near the needle insertion site at the stomach, where adenocarcinomas rather than ESCC usually arise [24]. The reported results from Furihata et al. show very low successful rates of orthotopic tumor formation (1/6) after most of the tumors regressed at the endpoint, whereas all four ESCC cell lines tested in our current model robustly and consistently showed successful formation of orthotopic tumors with fewer cell numbers inoculated in smaller volumes.

Notably, our previous studies indicated KYSE150 luc requires 1 × 10^6^ cells for subcutaneous inoculation to obtain 100% tumorigenicity in nude mice. The subcutaneous tumors obtained were frequently ulcerated in the central portion of the tumor and often showed cyst-like properties as the subcutaneous approach could only model tumor growth and local invasion related to the ectopic anatomical context of the skin mesenchyme [46]. On the other hand, orthotopic tumors derived from the KYSE150 luc cell line produced solid tumors and required only one-tenth the inoculum of cells (1 × 10^5^ cells) to achieve 100% tumorigenicity. Most importantly, macroscopic tissue necrosis or cyst-like properties were not observed in the orthotopic model tumors. The current ESCC orthotopic model provides a more optimal tissue microenvironment, including stromal cells, lymphatic and vascular vessels, and the innate immune system, necessary for ESCC* in situ* growth.

Histopathological examinations of the orthotopic tumors indicate invasion from mucosa to adventitia with lymphovascular permeation in the mouse esophagus. Tumor cells were observed in mucosa, submucosa, muscles, and adventitia. Furthermore, nodular lesions observed in orthotopic tumors highly resemble those found in human tumors. Perineural infiltration by squamous cell carcinoma is another commonly observed characteristic in ESCC and was also detected in our orthotopic tumors. Presence of perineural infiltration in squamous cell carcinoma was reported to be significantly correlated with cancer TNM stage, lymph node metastases, tumor grade, and depth of tumor infiltration, which can potentially serve as a significant independent prognosis marker for poor overall survival [36]. This orthotopic model, thus, recapitulates various human clinical pathological features commonly found in ESCC, including esophageal luminal stricture, lymphovascular invasion, adventitia invasion, perineural infiltration, and nodular tumor lesions, which strongly resemble the human ESCC tumor-stromal microenvironment. Compared to subcutaneous models, orthotopic models better recapitulate metastasis with sufficient penetrance and reproducibility and are useful for predicting the clinically relevant drug dosages [19, 46].

We tested different cell numbers of the KYSE150 cells during pilot studies, but since 1 × 10^5^ cells produced consistent tumor sizes and survival times for other cells, then the other cell numbers were not tested for KYSE150. A range of cell numbers are reported that all result in 100% tumorigenicity for the four ESCC cell lines tested (81-T, SLMT-1, KYSE30, and KYSE150). The range utilized was from 5 × 10^4^ to 5 × 10^5^ cells. Depending on the need of the study, investigators have some flexibility about choice of cell numbers to utilize in their studies.

The usefulness of this current esophageal orthotopic model as a platform for molecular cancer studies was further demonstrated by silencing of the* AKT* gene, which is known to play a critical role in ESCC tumorigenesis by regulating invasion, angiogenesis, and metastasis [31, 47]. The* in vivo* orthotopic tumor growth, as monitored by bioluminescence, showed a statistically significant tumor growth inhibition after AKT knockdown. An* in vitro* inhibitory effect upon real-time invasion assay was also observed for AKT knockdown.

The current surgical orthotopic ESCC model faithfully mimics the human clinical pathogenesis of ESCC development. The mutational landscape of ESCC involves multiple fundamental pathways including p53, PI3K/AKT, Wnt, and Notch pathways [48, 49]. These mutations may result in deregulation of signaling networks intracellularly and pathway activation disrupting the normal cell-cell and cell-matrix interaction in the tumor microenvironment that leads to ESCC tumorigenesis and metastasis. The newly established ESCC orthotopic tumor model described now provides the ideal platform for ESCC basic and preclinical research performed under conditions closely mimicking the expected* in situ* tumor microenvironment and possible examination of key features of advanced ESCC including local invasion and metastasis. Furthermore, the current ESCC orthotopic model will allow rapid preclinical drug testing and translational study of the potentially druggable alterations such as* PIK3CA, EGFR* amplification, and others from over one hundred candidates recently identified by the genomic sequencing studies carried out in ESCC [48, 49]. The orthotopic mouse models hold promise for the potential use for testing combinatorial therapies, new therapeutic targets, and systemic effects of treatments with high reproducibility [50]. We expect this newly established orthotopic model to provide a versatile and robust system for future ESCC studies and to have therapeutic application in pharmacokinetics and pharmacodynamics drug discovery.

## 5. Conclusion

This ESCC orthotopic mouse model allows investigation of gene functions of cancer cells in a more natural tumor microenvironment and has advantages over previously established models. It provides a versatile platform with potential application for metastasis and therapeutic regimen testing.
